# Children's social preference for teachers versus peers in autism inclusion classrooms: An objective perspective

**DOI:** 10.1002/aur.3276

**Published:** 2024-12-03

**Authors:** Madison Drye, Chitra Banarjee, Lynn Perry, Alyssa Viggiano, Dwight Irvin, Daniel Messinger

**Affiliations:** ^1^ Department of Psychology University of Miami Miami Florida USA; ^2^ College of Medicine University of Central Florida Orlando Florida USA; ^3^ Anita Zucker Center for Excellence in Early Childhood Studies University of Florida Gainesville Florida USA; ^4^ Department of Electrical and Computer Engineering University of Miami Miami Florida USA; ^5^ Department of Pediatrics University of Miami Miami Florida USA; ^6^ Department of Music Engineering University of Miami Miami Florida USA

**Keywords:** autism spectrum disorder, developmental disabilities, inclusion classrooms, objective measurement, social interaction

## Abstract

In inclusive preschools, children with autism spectrum disorder (ASD) and other developmental disabilities (DD) are less socially engaged with peers than are typically developing (TD) children. However, there is limited objective information describing how children with ASD engage with teachers, or how teacher engagement compares to engagement with peers. We tracked over 750 hours' worth of children's (*N* = 77; *N*_ASD_ = 24, *N*_DD_ = 23, *N*_TD_ = 30; *M*_age_ = 43.98 months) and teachers' (*N* = 12) locations and orientations across eight inclusion preschool classrooms to quantify child‐teacher and child‐peer social preference. Social approach velocity and time in social contact were computed for each child and compared across social partners to index children's preference for teachers over peers. Children with ASD approached teachers–‐but not peers—more quickly than children with TD, and children with ASD were approached more quickly by teachers and more slowly by peers than children with TD. Children with ASD spent less time in social contact with peers and did not differ from children with TD in their time in social contact with teachers. Overall, children with ASD showed a greater preference for approaching, being approached by, and being in social contact with teachers (relative to peers) than children with TD. No significant differences emerged between children with DD and children with TD. In conclusion, children with ASD exhibited a stronger preference for engaging with teachers over peers, re‐emphasizing the need for classroom‐based interventions that support the peer interactions of children with ASD.

## Lay Summary

We used automatic tracking of children's movement to ask whether children with autism spectrum disorder (ASD) engage differently with peers and teachers than children with typical development in inclusion preschool classes. Compared to their peers with typical development, children with ASD preferred teachers over peers, and teachers were quicker to approach children with ASD. This suggests the need for continued efforts by teachers to foster peer interaction for children with ASD in inclusion preschool classrooms.

## INTRODUCTION

Inclusion classrooms, in which children with autism spectrum disorder (ASD) and other developmental disabilities (DD) are educated alongside their typically developing (TD) peers, can be a best practice intervention context for promoting interaction and social learning. Adhering to the Individuals with Disabilities Education Act, inclusion classrooms strive to promote the integration of children with disabilities into mainstream education settings, fostering their social development through provision of opportunities to interact with others (Hayes & Bulat, 2017).

Peers play a crucial role in shaping the social experiences of children with ASD in inclusion classrooms. Research has consistently highlighted the importance of early childhood interactions, particularly those with peers, in laying the foundation for social skills and academic success (Jones et al., 2015; Santos & Vaughn, 2018). Inclusion classroom settings are designed to promote the development of social skills for children with ASD through increased opportunities for social interaction with TD peers (Guralnick & Bruder, 2016). Nevertheless, for children with ASD, navigating, forming, and maintaining relationships with peers may be challenging due to the difficulties they face with social interaction (American Psychiatric Association, 2022).

Homophily, the tendency for individuals to interact with peers who are similar to themselves, affects preferences in social interaction. Homophily with regard to children's language ability, gender, and academic achievement have been shown to influence classroom social dynamics (DeLay et al., 2016; Messinger et al., 2019; Smirnov & Thurner, 2017). Chen et al. (2019) documented homophily with respect to disability status in a study of teacher‐reported social engagement in inclusive classroom settings. Teachers reported more social interaction within concordant dyads (DD‐DD, TD‐TD) than discordant dyads (TD‐DD; Chen et al., 2019). Likewise, Fasano et al. (2021) found limited interaction between children with ASD and their TD peers. The extant evidence suggests that despite intentional integration in the classroom, children with ASD and typical development interact less frequently than other pairs of children.

Preschool teachers play a key role in shaping social dynamics within inclusive settings, particularly with respect to bridging potential interaction barriers between children with ASD and children with TD. Yet, research on the direct impact of teacher actions on peer interaction for children with ASD has yielded mixed findings. Some studies show that adult scaffolding can be effective in facilitating peer social interaction for children with ASD (Feldman & Matos, 2013). Conversely, the amount of time preschoolers spend with teachers may be negatively associated with their time spent interacting with peers (Harper & McCluskey, 2003). Moreover, teachers' intentional facilitation of peer interaction may be relatively uncommon in the classroom setting (Hong et al., 2020; Hume et al., 2019). While teachers are crucial for fostering peer social interactions in inclusive preschool settings, high levels of interaction with teachers may inadvertently limit opportunities for peer engagement, particularly among children with DD such as ASD. These seemingly contradictory findings indicate a need for a more nuanced understanding of teacher involvement with children with ASD.

### Capturing the dynamics of social interaction

Central to understanding the social experiences of children with ASD in inclusive classrooms is social motivation. Chevallier et al. (2012) suggested that individuals with ASD may show a diminished preference for engaging in social interactions compared to their TD peers. This reduced social motivation may be manifested in how children with ASD approach and interact with peers, their interest in social activities, and their ability to maintain social interactions (Chevallier et al., 2012). Thus, discrepancies in social motivation may influence who children with ASD prefer to interact with. Applying this theory to the inclusive preschool setting prompts us to investigate social preference in the context of children's interactions with a diverse group of peers and teachers.

Despite the importance of social preference in understanding the social experiences of children with ASD, operationalizing preferential engagement between children and teachers has posed significant challenges. To date, research in preschool settings has largely relied on observational approaches (live or video), such as teacher reports and manual coding of observations, to investigate how children interact with their social partners (Irvin et al., 2017). However, these methods may not fully capture the large‐scale differences in social preference in naturalistic settings.

Wearable sensor technology offers a promising solution to capturing social preference. Wearable devices, such as those used for location tracking, enable automated, fine‐grained data collection, potentially leading to a comprehensive picture of social preference and the mechanisms that motivate it (Elbaum et al., 2024; Gonzalez Villasanti et al., 2020; Irvin et al., 2018). Over the past decade, the use of sensing technology has become more common in the preschool space and has a multitude of advantages over traditional measurement approaches (e.g., objective, can capture the behaviors of many individuals at the same time, no reliability checks needed; Elbaum et al., 2024; Foster et al., 2024). Interpersonal proximity, a key constituent of social interaction, can serve as an index of positive affiliation within the preschool classroom (Horn et al., 2024; Howes, 1990; Irvin et al., 2021; Santos & Vaughn, 2018). By tracking proximity and social contact, the duration of that contact, and the overall speed with which children approach their social partners, wearable sensors can quantify social affiliation and may shed light on children's motivation to engage with specific partners. Variables derived from location tracking—often in collection with other measures—show initial promise as measures of children's social engagement (Altman et al., 2020; Banarjee et al., 2023). An objective measure of social contact derived from wearable sensors, for example, was negatively associated with older children's ratings of their own loneliness (Tsou et al., 2024). Likewise, Banarjee et al. (2023) found that children in concordant dyads (e.g., ASD‐ASD) exhibited higher social approach velocities and spent more time in social contact compared to discordant dyads (e.g., ASD‐TD), underscoring the relevance of homophily in inclusion preschool classrooms. Moreover, two specific measures of social affiliation (social approach velocity and time in social contact) were significantly and positively associated with one another (Banarjee et al., 2023). Building on this foundation, the current study unites data from Banarjee et al. (2023) on peers with new data on children's engagement with teachers to understand social preference among children with and without ASD in inclusion preschool classrooms.

In this study, we investigate social interaction patterns in inclusive preschool settings, focusing on 77 children with ASD, DD, and TD as well as their 12 teachers. We employ an objective approach using automated, location‐based measurements to quantify social interactions. Specifically, we measure social approach velocity (how quickly children approach and are approached by teachers or peers) and time in social contact (length of time spent interacting). We hypothesize that children with ASD will exhibit different social approach patterns than TD children, particularly in their approach velocity toward teachers and teachers' approach velocity toward them, as well as their time in social contact with teachers. We then calculate social preference by examining differences in children's approach behaviors and contact duration with teachers versus peers. Similarly, we hypothesize that children with ASD will show a heightened preference for teacher engagement over peer engagement compared to children with TD. This study aims to provide an objective perspective on social engagement patterns during the preschool years and shed light on the role of real‐time preferential engagement with peers and teachers in shaping children's social experiences.

## METHODS

A total of 77 children (33 female) enrolled in inclusion preschool classrooms were included in this study (Banarjee et al., 2023). Child participants were 3 to 5 years of age (*M* = 43.98 months, SD = 7.31; see Table 1) and included 24 children with ASD, 23 children with other DD, and 30 children with TD (see Table 2). Children were Hispanic (66) and non‐Hispanic (11), and their races included White (74), Black (2), and multiracial (1). There were 12 teacher participants (all female; see Table 2), 9 of whom were Hispanic, and whose races were White (10) and Black (2). Each classroom consisted of one lead teacher and 1–3 paraprofessional teachers. Recruitment and procedures for this study were approved by the university's Institutional Review Board with an overall child consent rate of 95.1% and a teacher consent rate of 100%.

**TABLE 1:** Age and standardized language scores by eligibility group.

Eligibility group	Age (months)		AC		EC		Total language	
	*M*	SD	*M*	SD	*M*	SD	*M*	SD
TD	45.14	6.65	113.41	16.32	111.28	22.42	113.24	2.36
ASD	42.43	8.21	82.45	17.02	74.40	16.42	77.15	16.58
DD	43.26	11.59	92.18	16.54	82.82	18.65	86.68	17.32

*Note*: The Preschool Language Scales Fifth Edition (PLS‐5) was administered to each child in the beginning of the school year to assess language abilities. The PLS‐5 generates standard scores for a child's abilities in the areas of Auditory Comprehension (AC), Expressive Communication (EC), and overall Total Language. Six children did not receive the PLS‐5.

Abbreviations: ASD, autism spectrum disorder; DD, developmental disabilities; TD, typically developing.

**TABLE 2:** Sample distribution by classroom.

	Sample size (# female)					
Classroom	ASD	DD	TD	Total children	Total teachers	Observation month (school year)
1	4 (0)	0	8 (5)	12 (5)	3	Jan Feb Mar Apr May (18–19)
2	3 (0)	0	8 (5)	11 (5)	4	Jan Feb Mar Apr May (18–19)
3	4 (1)	0	7 (6)	11 (7)	3	Nov Dec Jan Feb Mar (19–20)
4	3 (1)	0	8 (7)	11 (8)	3	Nov Dec Jan Feb Mar (19–20)
5	3 (1)	7 (0)	1 (0)	11 (1)	3	Jan Mar Apr May (18–19)
6	1 (0)	4 (2)	1 (0)	6 (2)	2	Nov Dec Jan Feb (19–20)
7	4 (1)	6 (3)	10 (7)	20 (11)	2	Nov Dec Jan Feb (19–20)
8	2 (1)	6 (3)	2 (1)	10 (5)	2	Jan Feb (19–20)
Totals:	24 (5)	23 (8)	30 (20)	77 (33)	12	

*Note*: There were two classrooms that were split into AM and PM sessions. In these classrooms, children with ASD attended either the AM or the PM session. All other students (except for one child) remained in the classroom for both AM and PM sessions. Similarly, all teachers were present for both AM and PM sessions in these classroom (except for one teacher). Four teachers remained in the same classroom for two consecutive years of data collection, resulting in 12 unique teachers. Teachers were all female.

Abbreviations: ASD, autism spectrum disorder; DD, developmental disabilities; TD, typically developing.

Participating children and their teachers were observed in eight inclusion preschool classrooms between 2018 and 2020 (see Table 2). The mean number of participating children in each classroom was 11.5 (SD = 3.9). Each child was observed on an average of 3.5 occasions (SD = 1.2), with each observation spanning the full class day (excluding naps) with a mean duration of 120.87 minutes (SD = 48.71). The mean number of teachers in a classroom was 2.8 (SD = 0.7). Each teacher was observed on an average of 3.4 occasions (SD = 1.6), with each observation lasting a mean duration of 105.85 min (SD = 44.92). In total, 783.23 hours (46,994 minutes) of tracked child and teacher movement and orientation data were collected.

Classrooms followed a variety of inclusion models, including Learning Experiences and Alternative Program (LEAP; *n* = 4 classes), a reverse mainstream model (*n* = 3 classes), and a balanced inclusion model (*n* = 1 class). The LEAP model places an emphasis on facilitating peer interaction; other classroom models, while inclusive, do not employ a particular style of intervention. Typical classroom activities included both structured, teacher‐led activities (e.g., circle time) and unstructured activities (e.g., free play). LEAP classes consisted of morning and afternoon sessions in a single classroom, where all but one of the children with TD were enrolled in both the morning and afternoon sessions. Following Banarjee et al. (2023), determination of a child's eligibility group was based on classroom classification (e.g., dedicated ASD intervention classes) and information from their Individualized Education Programs (IEP), supplemented in two cases by parent report confirming an ASD diagnosis for a child with DD eligibility.

### Data collection

Ultra‐Wideband (UWB) Radio Frequency Identification (RFID) tracking facilitated automated and real‐time assessment of children's and teachers' location and orientation in the classroom. Classroom dimensions (in meters) were 8.97 × 8.86, 8.76 × 8.93, 9.58 × 8.70, and 8.39 × 11.12. Four radio cell sensors, linked by a network cable and positioned in the corners of the classrooms, relayed data to a dedicated laptop running Ubisense Location Recorder software. Using the Ubisense Tag Module Research Dimension4 with Research Upgrade system, receivers in the corners of each classroom received UWB‐RFID signals at 2–4 Hz allowing continuous measurement of location with a 15 cm spatial accuracy (Barbieri et al., 2021; Irvin et al., 2018; Matakos et al., 2017). During observations, participants wore vests (children), or fanny packs (teachers) embedded with active RFID tags, which were tracked by corner‐mounted sensors. Real‐time tag location was determined by means of triangulation (angle of arrival, AoA) and time differences in arrival (TDoA). Each individual wore two tags (left and right) to provide information about their orientation (i.e., the direction being faced). The midpoint of the tags' XY coordinates was used to determine an individual's location, and measurements were collected at a rate of 10 times per second. Laboratory staff placed vests on consented children at the start of the school day, and the compliance rate for children tolerating the wearable vest for a given observation was high (>97%).

### Measures

For each observation period, participants' tracked locations and orientations were used to compute and visualize their movement throughout the school day. We leveraged automated measures of preschoolers' and teachers' positions and orientations in the classroom to compute social approach velocity and time in social contact between each participant (Helbing, 2001). Children participated in a range of activities (e.g., group and individual; structured and unstructured) throughout the day.

Social approach involves both an agent (the approacher) and a partner (the child or teacher being approached). Social approach velocity is the mean distance traveled per tenth of a second by the approacher toward the position of each partner in the previous tenth of a second, weighted by the pair's initial distance, orientation, and angle of movement. Specifically, social approach velocity is operationalized as the proportion of the initial distance between two social partners traveled by the approacher per tenth of a second. Time in social contact is the proportion of time a child spent in social contact with a partner over the total time both partners were present in the classroom. Social contact is defined as moments when both partners co‐locate between 0.2‐ and 2‐meters distance of one another and are mutually oriented to each other at less than or equal to |45°| (see Table 3). The distance criterion for detecting social interaction is determined by a radial distribution function, g(r), indicating when pairs co‐locate at closer distances than expected by chance (Banarjee et al., 2023). Here, chance refers to the probability of two individuals being at a given distance given a null model of subjects' random movement in the classroom irrespective of the positions of others (Messinger et al., 2019; Rozenfeld et al., 2010).

**TABLE 3:** Descriptions and equations (if applicable) for each measure of interest.

Measure	Description	Equation (if applicable)
Linear velocity	The change in an individual's location over time. Location is based on the midpoint of each subject's left (L) and right (R) RFID tags	Linear Velocity $\left[t\right]=\sqrt{{\left(x\left[t\right]-x\left[t-1\right]\right)}^{2}+{\left(y\left[t\right]-y\left[t-1\right]\right)}^{2}}$, where $x\left[t\right]=\frac{1}{2}\left(x_{R}\left[t\right]+x_{L}\left[t\right]\right)$ and $y\left[t\right]=\frac{1}{2}\left(y_{R}\left[t\right]+y_{L}\left[t\right]\right)$
Orientation: Partner A to Partner B	The orientation (θ) of the approacher to the partner being approached. Orientation is based on the coordinates of each subject's left (L) and right (R) RFID tags	*Orientation* $\text{θ[t‐1]=atan}2\left(\left(x_{R}\left[t-1\right]-x_{L}\left[t-1\right]\right),\left(y_{L}\left[t-1\right]-y_{R}\left[t-1\right]\right)\right)$ and *Subsequent orientation* $\text{θ[t]=atan}2\left(\left(x_{R}\left[t\right]-x_{L}\left[t\right]\right),\left(y_{L}\left[t\right]-y_{R}\left[t\right]\right)\right)$
Approach distance: Partner A to Partner B	The distance each child or teacher (A) moved toward the initial position of their partner (B). Negative approach distance (i.e., moving away from partner B) was not analyzed here	*Approach*_*A→B*_ *[* t *] = Distance*_*A → B*_ *[* t−1 *]* − *Subsequent Distance*_*A→B*_ *[* t *],* where *Distance*_*A → B*_ $[t‐1]=\sqrt{{\left(x_{A}\left[t-1\right]-x_{B}\left[t-1\right]\right)}^{2}+{\left(y_{A}\left[t-1\right]-y_{B}\left[t-1\right]\right)}^{2}}$ and *Subsequent Distance*_*A→B*_ $[t]=\sqrt{{\left(x_{A}\left[t\right]-x_{B}\left[t\right]\right)}^{2}+{\left(y_{A}\left[t\right]-y_{B}\left[t\right]\right)}^{2}}$
Social approach velocity	The distance traveled per tenth of a second by the approacher (A) toward the initial position of each partner (B), weighted by initial distance, orientation of the approacher ($\theta_{o}$), and angle of movement of the approacher ($\theta_{m}$)	Social Approach_Weighted_ $\left[t\right]=\frac{{\text{Approach}}_{A\to B}\left[t\right]∙\mathrm{co}s^{2}\left(\theta_{o}\left[t-1\right]\right)∙\mathrm{co}s^{2}\left(\theta_{m}\left[t\right]\right)}{\;{\text{Distance}}_{A\to B}\left[t-1\right]}$
Social approach velocity: Child to teacher/peer	The mean distance traveled per tenth of a second by the Approacher (child; A) toward the initial position of each partner (teacher/peer; B), weighted by initial distance, orientation ($\theta_{o}$), and angle of movement ($\theta_{m}$)	
Social approach velocity: Teacher/Peer to child	The mean distance traveled per tenth of a second by each approacher (teacher/peer; A) toward the initial position of the child (B), weighted by initial distance, initial orientation ($\theta_{o}$), and angle of movement ($\theta_{m}$)	
Social approach preference: Teacher over peer	Child as approacher (A): The mean difference between the velocity with which a child approaches their teachers minus the velocity with which a child approaches their peers. This is the relative velocity toward teachers (versus peers) Child being approached (B); The mean difference between the velocity with which a child is approached by their teachers minus the velocity with which the child is approached by their peers. This is the relative velocity of teachers' (versus peers) social approach toward the child	
Time in social contact with teachers/peers	The mean proportion of shared classroom time that a child spends in social contact with their teachers/peers	
Social contact preference: Teacher over peer	The mean difference of a child's proportion of shared classroom time spent with teachers minus proportion of classroom time spent with peers	

For each child, we determined the child's social approach velocity toward teachers, and teachers' social approach velocity toward the child. These values were then compared to the individual child's parallel social approach values with peers. The difference between teacher and peer velocities (teacher minus peer) yielded a measure of the child's preference for approaching teachers over peers, and the preference of teachers over peers for approaching the child. Lastly, we determined the child's proportion of classroom time spent in social contact with teachers, which was compared to the child's time in social contact with peers to generate a difference that indexed the child's preference for time in social contact with teachers over peers (see Table 3). All results involving peers only are based on analyses of data reported by Banarjee et al. (2023). Unlike in Banarjee et al. (2023), we focused exclusively on effects of the child's group (e.g., ASD) and did not include a homophily term (i.e., presence of a match between child and partner's groups) in these models.

### Mixed‐effects models

Mixed‐effects (multilevel) modeling was conducted in R (R Core Team, 2023). Models were fit and compared using the lmer function in the “lme4” package. Models employed restricted maximum likelihood (REML) estimation methods for parameterization and model comparison (Bates et al., 2015). Observations (Level‐1) were nested in children (Level‐2) which were nested in classrooms (Level‐3). Effect sizes were estimated using the lme. dscore function in the “EMATools” package (Kleiman, 2021). The significance test of fixed effects was performed using the Wald test (Bates et al., 2015). All models contained a random intercept at the child‐ and classroom‐level. The significance of random effects was estimated by comparing models with and without the corresponding random effect, where differences in model deviance [−2*(Log Likelihood)] were distributed as chi‐square. Between‐subjects effects were parameterized as a TD intercept with ASD and DD group contrasts. Continuous predictor variables were mean centered at the child level (Enders & Tofighi, 2007). As this research was exploratory, we determined significance using a conservative alpha level of 0.01.

## RESULTS

### Child‐teacher social approach velocity

Mixed‐effects models predicted the velocity of children's social approach toward teachers, as well as the velocity of teacher's social approach toward children. In both models, children with TD were the reference group so that the ASD and DD terms parameterize the observed difference in social approach velocity for each eligibility group compared to the TD group. In the first model, children with ASD approached teachers at higher velocities than their TD peers, while children with DD did not differ from children with TD in their social approach velocity toward teachers (*p*_ASD_ <0.001, *d*_ASD_ = 1.00; *p*_DD_ = 0.057, *d*_DD_ = 0.37; see Table 4A and B). Parallel results emerged in the second model predicting teacher approach velocities toward children. Children with ASD were approached by teachers at higher velocities than their TD peers, whereas children with DD did not differ from children with TD in the velocity with which their teachers approached them (*p*_ASD_ <0.001, *d*_ASD_ = 1.12; *p*_DD_ = 0.125, *d*_DD_ = 0.31; see Table 5A and B). Overall, children with ASD approached and were approached by teachers at higher velocities than their TD peers were.

**TABLE 4a:** Null model for predicting child to teacher social approach.

Fixed effects						
Group	*B*	SE	95% CI	*t*	*p*	*d*
(intercept)	0.0025	0.00018	0.0022–0.0029	13.98	<0.001	

Random Effects	
σ^2^	3.14 × 10^−7^
τ_00 subject: classroom_	9.68 × 10^−8^
τ_00 classroom_	2.45 × 10^−7^
ICC_subject_	0.15
ICC_classroom_	0.37

**TABLE 4b:** Predicting child to teacher social approach.

Fixed effects						
Group	*B*	SE	95% CI	*t*	*p*	*d*
TD (intercept)	0.0024	0.00019	0.0020–0.0027	12.40	<0.001	
ASD versus TD	0.00047	0.00010	0.00027–0.00067	4.57	**<0.001**	1.00
DD versus TD	0.00026	0.00014	−0.00001–0.00053	1.91	0.057	0.37

Random Effects	
σ^2^	3.12 × 10^−7^
τ_00 subject: classroom_	6.34 × 10^−8^
τ_00 classroom_	2.51 × 10^−7^
ICC_subject_	0.10
ICC_classroom_	0.40

Abbreviations: ASD, autism spectrum disorder; DD, developmental disabilities; TD, typically developing.

**TABLE 5a:** Null model for predicting teacher to child social approach.

Fixed effects						
*Group*	*B*	*SE*	*95% CI*	*t*	*p*	*d*
(intercept)	0.0028	0.00023	0.0022–0.0030	12.00	<0.001	

Random effects	
σ^2^	3.46 × 10^−7^
τ_00 subject: classroom_	6.46 × 10^−8^
τ_00 classroom_	4.05 × 10^−7^
ICC_subject_	0.08
ICC_classroom_	0.50

### Child‐peer social approach velocity

Mixed‐effects models predicted the velocity of children's social approach toward peers, as well as the velocity of peers' social approach toward children. ASD and DD terms parameterized the observed difference in social approach velocity for each eligibility group compared to the TD group. In the first model, there were no significant differences in the velocity with which children approached their peers (*p*_ASD_ = 0.043, *d*_ASD_ = −0.44; *p*_DD_ = 0.58, *d*_DD_ = 0.11; see Table 6A and B). However, children with ASD were approached more slowly by their peers than children with TD (*p*_ASD_ = 0.006, *d*_ASD_ = −0.31). No differences were observed between peers' approach velocities toward children with DD and children with TD (*p*_DD_ = 0.73, *d*_DD_ = −0.04; see Table 7A and B).

**TABLE 5b:** Predicting teacher to child social approach.

Fixed effects						
Group	*B*	SE	95% CI	*t*	*p*	*d*
TD (intercept)	0.0026	0.00022	0.0022–0.0030	11.74	<0.001	
ASD versus TD	0.00047	0.000090	0.00028–0.00065	4.94	**<0.001**	1.12
DD versus TD	0.00020	0.00013	−0.000050 – 0.00045	1.54	0.125	0.31

Random effects	
σ^2^	3.45 × 10^−7^
τ_00 subject: classroom_	3.24 × 10^−8^
τ_00 classroom_	3.55 × 10^−7^
ICC_subject_	0.04
ICC_classroom_	0.49

Abbreviations: ASD, autism spectrum disorder; DD, developmental disabilities; TD, typically developing.

**TABLE 6a:** Null model predicting child to peer social approach.

Fixed effects						
Group	*B*	SE	95% CI	*t*	*p*	*d*
(intercept)	0.0033	0.00024	0.0028–0.0037	13.55	<0.001	

Random effects	
σ^2^	3.80 × 10^−7^
τ_00 subject: classroom_	1.96 × 10^−8^
τ_00 classroom_	4.46 × 10^−7^
ICC_subject_	0.02
ICC_classroom_	0.53

**TABLE 6b:** Predicting child to peer social approach.

Fixed effects						
Group	*B*	SE	95% CI	*t*	*p*	*d*
TD (intercept)	0.0033	0.00025	0.0028–0.0038	13.14	<0.001	
ASD versus TD	−0.00018	0.000090	−0.00036 – −0.000010	−2.03	0.043	−0.44
DD versus TD	0.000070	0.00012	−0.00017 – 0.00031	0.56	0.576	0.11

Random effects	
σ^2^	3.81 × 10^−7^
τ_00 subject: classroom_	1.22 × 10^−8^
τ_00 classroom_	4.67 × 10^−7^
ICC_subject_	0.01
ICC_classroom_	0.54

Abbreviations: ASD, autism spectrum disorder; DD, developmental disabilities; TD, typically developing.

**TABLE 7a:** Null model predicting peer to child social approach.

Fixed effects						
Group	*B*	SE	95% CI	*t*	*p*	*d*
(intercept)	0.0033	0.00024	0.0028–0.0038	13.49	<0.001	

Random effects	
σ^2^	2.93 × 10^−7^
τ_00 subject: classroom_	1.37 × 10^−24^
τ_00 classroom_	4.57 × 10^−7^
ICC_subject_	0.00
ICC_classroom_	0.61

### Teacher (versus peer) social approach preference

The difference between teacher social approach velocity and peer social approach velocity was conceptualized as an index of a child's preference for engaging with teachers over peers when the child is the approaching partner, and a preference of teachers over peers for approaching a target child. Two mixed‐effects models predicted these preference indices. In the first model, the eligibility group of the approacher (the child) was used as the primary predictor, and the second model used the eligibility group of the child being approached as the primary predictor.

**TABLE 7b:** Predicting peer to child social approach.

Fixed effects						
Group	*B*	SE	95% CI	*t*	*p*	*d*
TD (intercept)	0.0033	0.00024	0.0028–0.0038	13.61	<0.001	
ASD versus TD	−0.00020	0.000070	−0.00035–−0.000060	−2.74	**0.006**	−0.31
DD versus TD	−0.000040	0.00010	−0.00024–0.00017	−0.34	0.732	−0.04

Random effects	
σ^2^	2.88 × 10^−7^
τ_00 subject: classroom_	0.00
τ_00 classroom_	4.54 × 10^−7^
ICC_subject_	0.00
ICC_classroom_	0.61

Abbreviations: ASD, autism spectrum disorder; DD, developmental disabilities; TD, typically developing.

Compared to children with TD, children with ASD showed an elevated preference for approaching their teachers over their peers (*p*_ASD_ <0.001, *d*_ASD_ = 1.19). Conversely, children with DD did not differ from children with TD in their preference for approaching teachers over peers (*p*_DD_ = 0.053, *d*_DD_ = 0.39; see Table 8A and B). In the second model with the child as the partner being approached, children with ASD were more preferentially approached by teachers over peers compared to the TD group (*p*_ASD_ <0.001, *d*_ASD_ = 1.19 ). Children with DD did not significantly differ from children with TD in terms of approach preference by teachers over peers (*p*_DD_ = 0.124, *d*_DD_ = 0.3; see Table 9A and B).

**TABLE 8a:** Null model predicting children's preference for approaching teachers over peers.

Fixed effects						
Group	*B*	SE	95% CI	*t*	*p*	*d*
TD (intercept)	−0.00069	0.00014	−0.0012–−0.00068	−5.11	<0.001	

Random effects	
σ^2^	3.97 × 10^−7^
τ_00 subject: classroom_	1.74 × 10^−7^
τ_00 classroom_	1.18 × 10^−7^
ICC_subject_	0.25
ICC_classroom_	0.17

Abbreviation: TD, typically developing.

**TABLE 8b:** Predicting children's preference for approaching teachers over peers.

Fixed effects						
Group	*B*	SE	95% CI	*t*	*p*	*d*
TD (intercept)	−0.00095	0.00013	−0.0012–−0.00068	−7.09	<0.001	
ASD versus TD	0.00067	0.00012	0.00043–0.00091	5.51	**<0.001**	1.19
DD versus TD	0.00030	0.00015	−0.00–0.00060	1.94	0.053	0.39

Random effects	
σ^2^	3.96 × 10^−7^
τ_00 subject: classroom_	1.07 × 10^−7^
τ_00 classroom_	9.35 × 10^−8^
ICC_subject_	0.18
ICC_classroom_	0.16

Abbreviations: ASD, autism spectrum disorder; DD, developmental disabilities; TD, typically developing.

**TABLE 9a:** Null model predicting preferential approach by teachers over peers toward children.

Fixed effects						
Group	*B*	SE	95% CI	*t*	*p*	*d*
(intercept)	−0.00048	0.00029	−0.0011–0.000090	−1.65	0.100	

Random effects	
σ^2^	5.41 × 10^−7^
τ_00 subject: classroom_	1.30 × 10^−7^
τ_00 classroom_	6.59 × 10^−7^
ICC_subject_	0.10
ICC_classroom_	0.50

### Child‐teacher social contact

Mixed‐effects model predicted the proportion of shared classroom time children spent in social contact with their teachers. Neither children with ASD nor children with DD differed from children with TD in their proportion of classroom time spent in social contact with teachers (*p*_ASD_ = 0.133, *d*_ASD_ = 0.37; *p*_DD_ = 0.676, *d*_DD_ = 0.09; see Table 10A and B).

**TABLE 9b:** Predicting preferential approach by teachers over peers toward children.

Fixed effects						
Group	*B*	SE	95% CI	*t*	*p*	*d*
TD (intercept)	−0.00073	0.00028	−0.0013–−0.00018	−2.61	0.010	
ASD versus TD	0.00067	0.00012	0.00043–0.00091	5.49	**<0.001**	1.19
DD vesus TD	0.00025	0.00016	−0.000070–0.00057	1.54	0.124	0.30

Random effects	
σ^2^	5.39 × 10^−7^
τ_00 subject: classroom_	6.17 × 10^−8^
τ_00 classroom_	5.71 × 10^−7^
ICC_subject_	0.05
ICC_classroom_	0.49

Abbreviations: ASD, autism spectrum disorder; DD, developmental disabilities; TD, typically developing.

**TABLE 10a:** Null model predicting children's time in social contact with teachers.

Fixed effects						
Group	*B*	SE	95% CI	*t*	*p*	*d*
(Intercept)	0.040	0.0031	0.033–0.046	12.75	**<0.001**	

Random effects	
σ^2^	4.03 × 10^−4^
τ_00 subject: classroom_	0.00
τ_00 classroom_	6.51 × 10^−5^
ICC_subject_	0.00
ICC_classroom_	0.14

### Child‐peer social contact

Mixed‐effects model predicted the proportion of shared classroom time children spend in social contact with their peers. Compared to TD children, children with ASD spent significantly less classroom time in social contact with peers (*p*_ASD_ <0.001, *d*_ASD_ = −0.44). Children with DD did not differ from children with TD in their time in social contact with peers (*p*_DD_ = 0.059, *d*_DD_ = −0.22; see Table 11A and B).

**TABLE 10b:** Predicting children's time in social contact with teachers.

Fixed effects						
Group	*B*	SE	95% CI	*t*	*p*	*d*
TD (intercept)	0.038	0.0033	0.031–0.045	11.38	<0.001	
ASD versus TD	0.0042	0.0028	−0.0013 – 0.0097	1.51	0.133	0.37
DD versus TD	0.0015	0.0037	−0.0057 – 0.0087	0.42	0.676	0.09

Random effects	
σ^2^	4.01 × 10^−4^
τ_00 subject: classroom_	1.17 × 10^−6^
τ_00 classroom_	6.28 × 10^−5^
ICC_subject_	0.00
ICC_classroom_	0.14

Abbreviations: ASD, autism spectrum disorder; DD, developmental disabilities; TD, typically developing.

**TABLE 11a:** Null model predicting children's time in social contact with peers.

Fixed effects						
Group	*B*	SE	95% CI	*t*	*p*	*d*
(Intercept)	0.046	0.0029	0.040–0.051	15.93	<0.001	

Random Effects	
σ^2^	1.71 × 10^−4^
τ_00 subject: classroom_	4.26 × 10^−8^
τ_00 classroom_	6.07 × 10^−5^
ICC_subject_	0.00
ICC_classroom_	0.26

### Teacher (versus peer) social contact preference

The difference in the proportion of time a child spends in social contact with their teachers and their peers was conceptualized as an index of a child's preference for engaging with teachers over peers. A mixed‐effects model predicted this difference in social contact with teachers and their peers. Compared to children with TD, children with ASD exhibited an elevated preference for social contact with teachers over peers (*p*_ASD_ = 0.001, *d*_ASD_ = 0.75). Children with DD did not differ from children with TD in their preference for social contact with teachers over peers (*p*_DD_ = 0.182, *d*_DD_ = 0.27; see Table 12A and B).

**TABLE 11b:** Predicting children's time in social contact with peers.

Fixed effects						
Group	*B*	SE	95% CI	*t*	*p*	*d*
TD (intercept)	0.049	0.0031	0.042–0.055	15.52	<0.001	
ASD versus TD	−0.0069	0.0018	−0.010–−0.0034	−3.89	**<0.001**	−0.44
DD versus TD	−0.0046	0.0024	−0.0093–0.00017	−1.90	0.059	−0.22

Random Effects	
σ^2^	1.64 × 10^−4^
τ_00 subject: classroom_	8.00 × 10^−20^
τ_00 classroom_	6.72 × 10^−5^
ICC_subject_	0.00
ICC_classroom_	0.29

Abbreviations: ASD, autism spectrum disorder; DD, developmental disabilities; TD, typically developing.

**TABLE 12a:** Null model predicting children's preference for time in social contact with teachers over peers.

Fixed effects						
Group	*B*	SE	95% CI	*t*	*p*	*d*
TD (intercept)	−0.0062	0.0034	−0.013–0.00051	−1.82	0.07	

Random effects	
σ^2^	4.46 × 10^−4^
τ_00 subject: classroom_	5.18 × 10^−5^
τ_00 classroom_	7.45 × 10^−5^
ICC_subject_	0.09
ICC_classroom_	0.13

### Associations between measures of social affiliation

**FIGURE 1:**
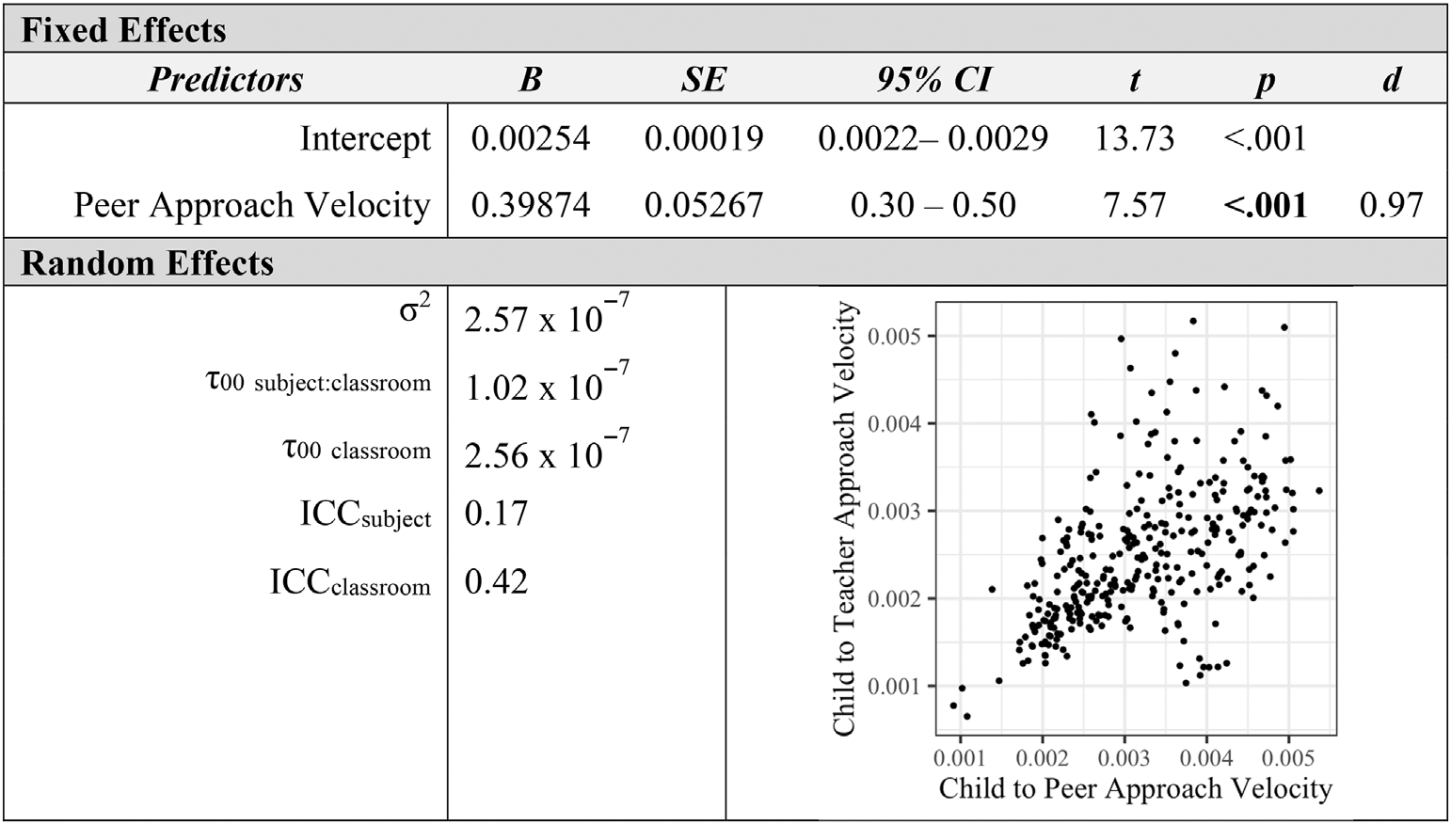
Child to peer social approach velocity as a predictor of child to teacher social approach velocity.

**FIGURE 2:**
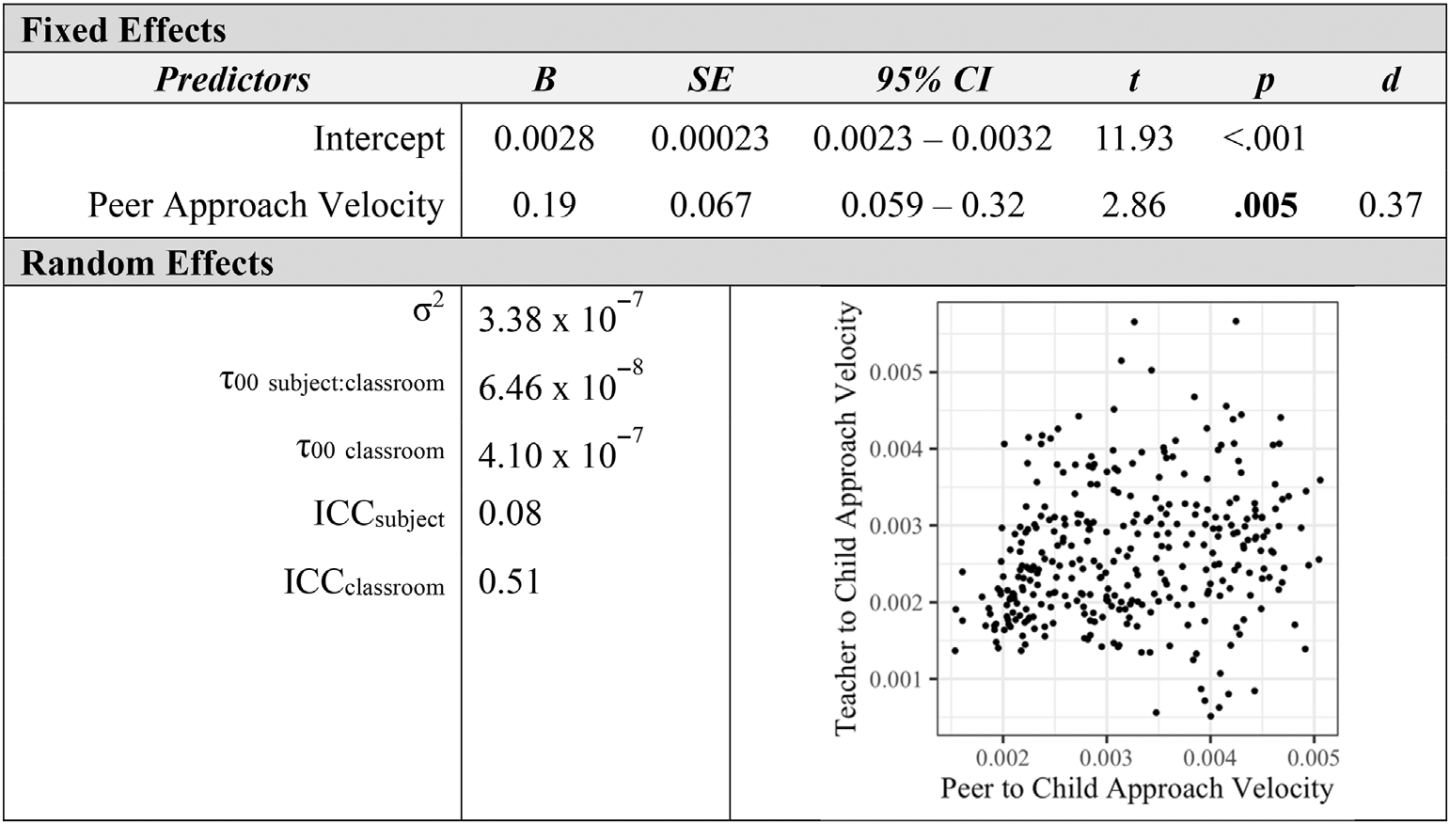
Peer to child social approach velocity as a predictor of teacher to child social approach velocity.

**FIGURE 3:**
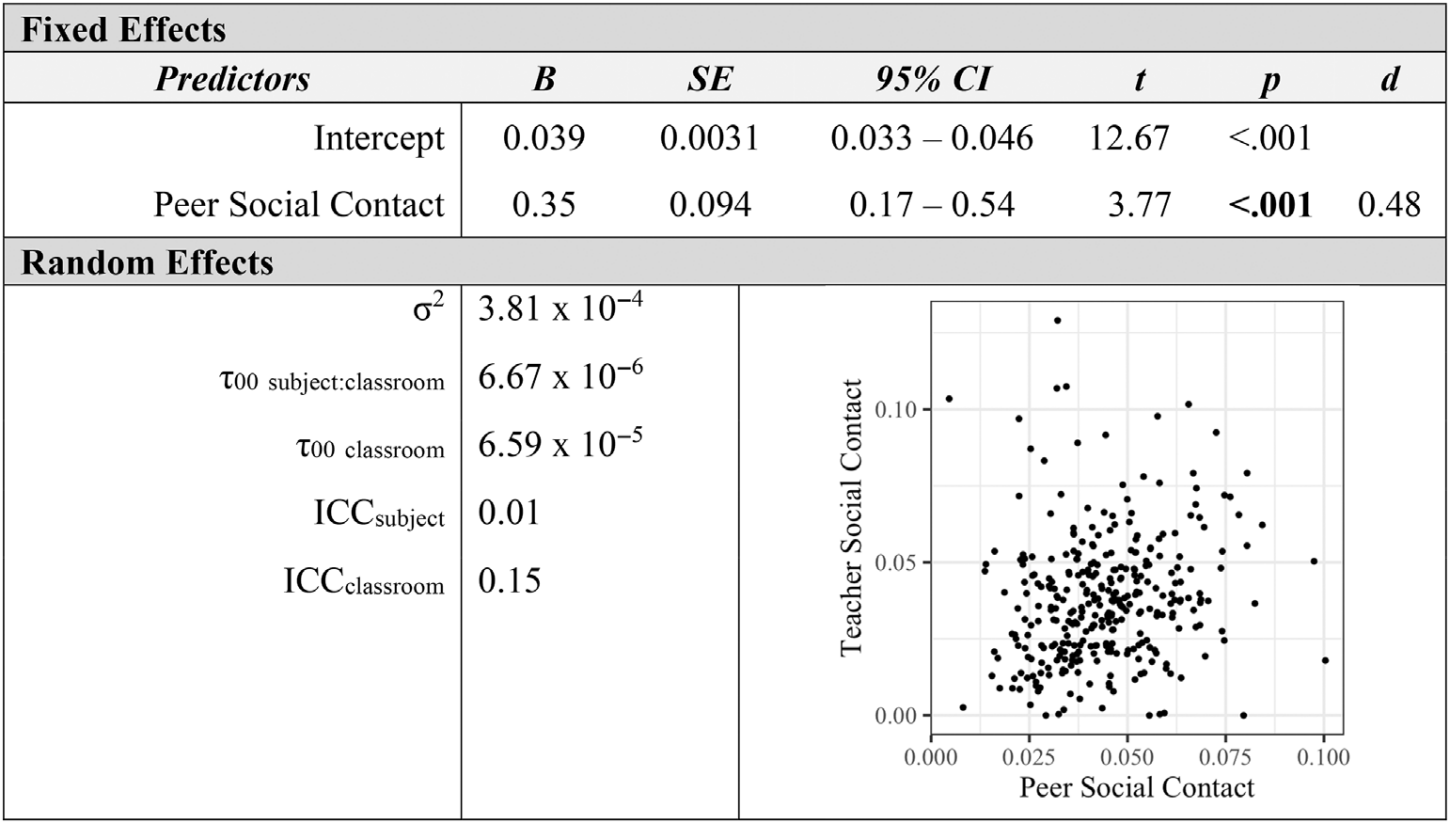
Peer social contact as a predictor of teacher social contact.

**FIGURE 4:**
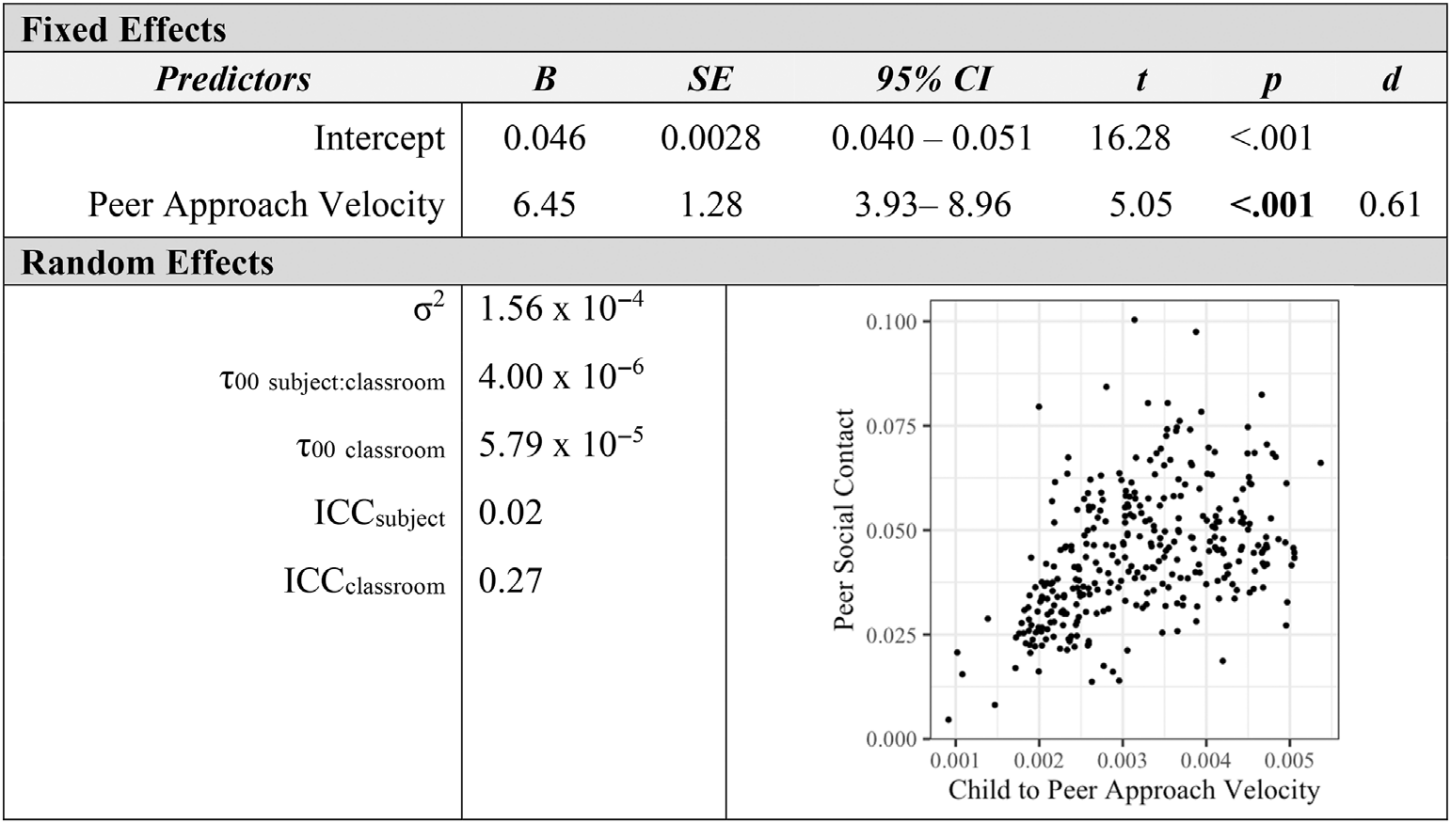
Child to peer social approach velocity as a predictor of peer social contact.

**FIGURE 5:**
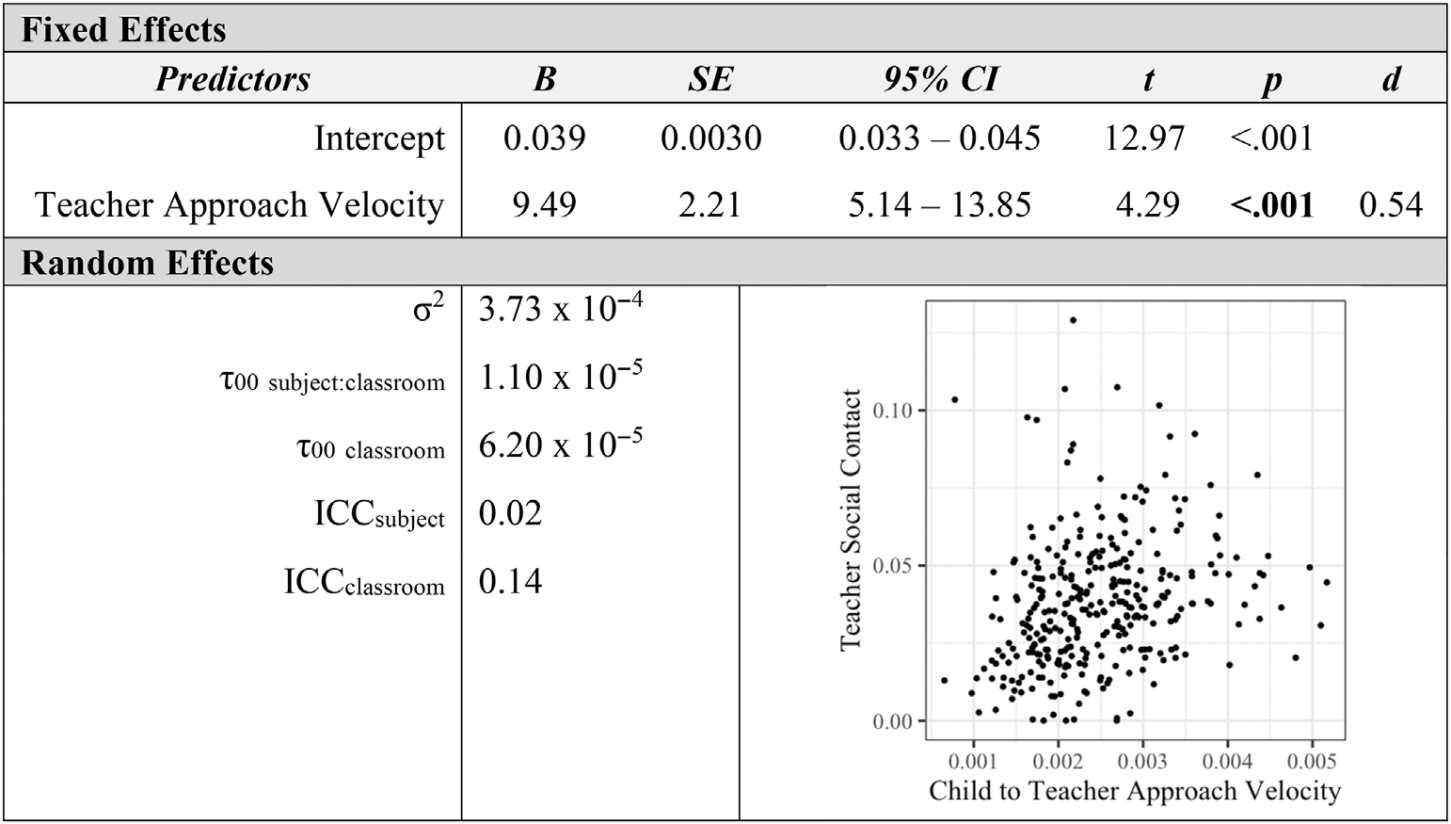
Child to teacher social approach velocity as a predictor of teacher social contact.

Additional mixed‐effects models examined how measures of affiliation with peers and teachers were associated at the individual level. Children's social approach velocity toward peers was positively associated with their approach velocity toward teachers in the classroom (*p* < 0.001, *d* = 0.97; see Figure 1). Similarly, peers' social approach velocity toward a child was a positive predictor of teachers' social approach velocity toward a child (*p* = 0.005, *d* = 0.37; see Figure 2). The proportion of classroom time children spent in social contact with peers was positively related to their social contact with teachers (*p* < 0.001, *d* = 0.48; see Figure 3). Finally, a child's social approach velocity was positively associated with time in social contact for both peer and teacher partners (*p*_peer_ <0.001, *d*_peer_ = 0.61; *p*_teacher_ <0.001, *d*_teacher_ = 0.54; see Figures 4 and 5). Supplementary analyses revealed no significant interaction effects between pairs of measures of social affiliation and eligibility group, though the number of predictors in these models suggest these null interaction results be interpreted with caution (see Figure S1). Overall, then, children's levels of social affiliation with peers were associated with their levels of social affiliation toward teachers—and the two measures of social affiliation toward peers were associated, as were the measures of social affiliation toward teachers.

**TABLE 12b:** Predicting children's preference for time in social contact with teachers over peers.

Fixed effects						
Group	*B*	SE	95% CI	*t*	*p*	*d*
TD (intercept)	−0.010	0.0038	−0.018–−0.0030	−2.76	0.006	
ASD versus TD	0.011	0.0033	0.0045–0.018	3.33	**0.001**	0.75
DD versus TD	0.0057	0.0043	−0.0027–0.014	1.34	0.182	0.27

Random Effects	
σ^2^	4.44 × 10^−4^
τ_00 subject: classroom_	3.61 × 10^−5^
τ_00 classroom_	7.72 × 10^−5^
ICC_subject_	0.06
ICC_classroom_	0.14

Abbreviations: ASD, autism spectrum disorder; DD, developmental disabilities; TD, typically developing.

### Child and classroom variance

Intraclass correlation (ICC) values were calculated to assess the variance explained at the child‐ and classroom‐levels for each model. ICC values were similar between null models (Tables 4a, 4b, 5a, 5b, 6a, 6b, 7a, 7b, 8a, 8b, 9a, 9b, 10a, 10b, 11a, 11b, 12a) and models containing fixed effects (Tables 4b, 5a, 5b, 6a, 6b, 7a, 7b, 8a, 8b, 9a, 9b, 10a, 10b, 11a, 11b, 12a, 12b). Across most models and variables, the subject‐level variance and resulting ICCs were consistently small relative to those at the classroom‐level. The exception to this trend was found in the model predicting children's preference for approaching teachers over peers, where individual differences and classroom effects seem to be contributing approximately equally to the overall variance in children's social preference behaviors. Overall, child‐level ICCs ranged from 0 to 0.18, indicating relatively low stability in measures of social behaviors. The highest ICC values, and thus the greatest levels of child‐level stability, were observed among measures of child‐initiated peer social behaviors (e.g., children approaching peers, children's approach preference for teachers over peers). Classroom ICCs, indicating classroom level stability, were low to moderately high with values ranging from 0.14 to 0.61. Greater classroom‐level stability was observed among measures of peer‐initiated social behaviors (e.g., peers approaching children, relative preference of teachers over peers for approaching a child).

## DISCUSSION

**FIGURE 6:**
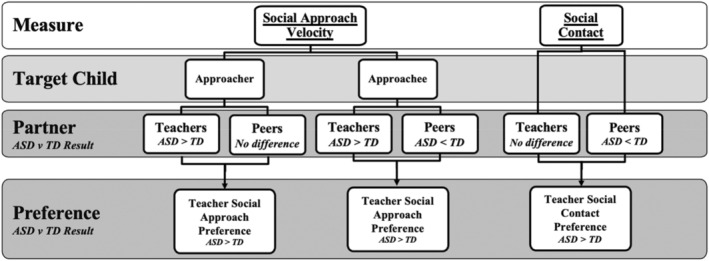
Overview of all measures and significant results. No significant differences were observed between TD and DD groups or DD and ASD groups for any measures (see Table S1). ASD, autism spectrum disorder; DD, developmental disabilities; TD, typically developing.

Inclusion classrooms offer unique environments where children with ASD spend considerable time alongside teachers and peers. This study leveraged objective data to explore social affinity among preschoolers with and without ASD in inclusion classrooms, focusing on their social preference for teachers over peers. We found converging evidence for children with ASD's social preference for teachers over peers relative to children with TD, as evidenced by a stronger preference for approaching, being approached by, and being in social contact with teachers over peers in the preschool classroom (see Figure 6 for a summary of all results). The inclination of children with ASD to prefer teachers in inclusive preschool settings may be reflect their histories of more successful interactions with teachers than peers, accentuated by heightened accessibility to teachers as a form of social support.

Notably, we observed no differences in any measures of social preference for children with DD compared to their TD counterparts. This finding suggests that the observed differences in preferential social engagement may be unique to ASD. This potential uniqueness could stem from the distinct social challenges faced by children with ASD, which may have a pronounced impact on social interaction in the preschool classroom. Further research is warranted to better understand social preference among children with different developmental profiles.

This study is a step forward in understanding the social dynamics of children with and without ASD using objective measurement. By leveraging two distinct but related metrics—social approach velocity and time in social contact—we captured key facets of social interaction. Given the facilitative role that interpersonal proximity plays in promoting children's development (Barnett et al., 2022), social approach velocity—which we hypothesize to be a proximity‐seeking behavior—sheds light onto preschoolers' propensities to initiate social interactions. Faster approaches may signify a greater drive for social engagement, reflecting both the approaching partner's social motivation and the approachability of the partner being approached. Complementarily, the assessment of time in social contact may index the extent to which individuals maintain their interactions, which may also reflect their social motivation. Taken together, these findings may offer insight into social motivation and preference patterns in the classroom.

The current study revealed heightened patterns of engagement between children with ASD and teachers in the inclusive classroom environment. First, children with ASD both approached and were approached by teachers at higher velocities than their TD peers, which may indicate the heightened presence of—and reliance on—rapid adult support for children with ASD. This heightened engagement could also reflect the preference of children with ASD for teachers as social partners due to a history of more successful interactions. However, while prior research suggests that rapid intervention from teachers may inhibit interactions with peers (Harper & McCluskey, 2003), the current study's data does not support this claim. Positive relationships were observed between social approach velocity to peers and teachers and time in social contact with both peers and teachers, suggesting that teacher engagement does not diminish peer interactions for children with ASD. Moreover, children with ASD did not differ from children with typical development in the degree to which they maintained social contact with their teachers. Despite differences in children's social approach velocity toward teachers, children with and without ASD engaged with their teachers for a similar proportion of their school day, which may reflect teachers' efforts to create an inclusive classroom atmosphere where all children receive similar amounts of teacher attention.

Although there were no group differences in social approach velocity when the child was the approaching partner, children with ASD spent less time engaged in social contact with their peers than TD children. These findings may reflect challenges with social motivation, particularly maintaining interactions with peers, experienced by children with ASD (Chevallier et al., 2012). These challenges may be compounded by children with ASD experiencing slower approach velocities from their peers than their TD counterparts, which appears to reflect decreased preference for interacting with classmates with ASD.

Our findings offer additional support for the notion that distinct patterns of physical movement can serve as indicators of social affiliation in the preschool classroom (Elbaum et al., 2024; Horn et al., 2024). We used wearable sensor technology to measure preschoolers' proximity with their social partners over time, which revealed group differences in how children with and without ASD engaged with their peers and teachers in the classroom. We found univariate measures of social affiliation—social approach velocity and time in social contact—to be positively associated with one another in this study. This consistency may offer evidence in support of using objectively measured social approach velocity and time in social contact as indicators of social affiliation or social motivation (Chevallier et al., 2012). Compared to traditional measures, which can be resource‐intensive and limited in scope, objective measures appear to have value in capturing continuous processes underlying social interaction (Elbaum et al., 2024; Foster et al., 2024).

### Implications for intervention

There is overwhelming agreement that meaningful inclusion goes beyond simply physically placing children with DD or ASD in a preschool classroom (Barton & Smith, 2015). Effective inclusion should involve the social integration and participation of all children, regardless of disability, in classroom activities and routines (Guralnick & Bruder, 2016; Sailor et al., 1990); however, discrepancies in social behaviors between children with ASD and TD peers may pose significant obstacles to fully harnessing the benefits that inclusion classrooms offer. To advance toward a goal of effective inclusion we need to: (1) better understand children's social preference, and (2) be able to provide teachers with data about the degree to which children with DD and ASD are engaging with others (Diamond et al., 2013; Irvin et al., 2017).

To assess social preference, we used a novel sensing technology that allows us to quantify the teacher and peer social preference of children with TD, DD, and ASD. Uniquely, it focuses on individual children, all measured at the same time, rather than the classroom as a whole (see Odom et al., 2011 for global measures of inclusion quality), allowing us to advance toward a more precise, meaningful measure of inclusion. Importantly, the current approach captures children's social preference in way that: (1) places little additional burden on teachers and could eventually be used to provide them with actionable data, and (2) could be used to evaluate classroom interventions aimed fostering preschoolers with ASD's engagement with peers and classroom adults. We found a positive association between children's measures of peer engagement and teacher engagement. Thus, information gathered about preschoolers' social preference can guide teachers in tailoring support strategies, which may include either facilitating peer interaction or directly engaging with a child. Future research into the timing and sequence of children initiating interactions with peers and teachers may help clarify whether teacher responsiveness is contributing positively to social opportunities for children with ASD, or if it may, in some cases, be counterproductive.

### Limitations, future directions, and conclusions

The current sample (77 children and 12 teachers) is relatively small, but teachers and children were observed on multiple occasions in eight classrooms. Dense longitudinal data–over 750 hours of child and teacher movement data (*M* = >8 h per person)–are useful for understanding children's everyday experiences (DeBolt et al., 2020; Perry et al., 2018; Roy et al., 2015). Moreover, our use of mixed effects models separately partitioned classroom‐ and individual‐level variance, and we adopted a conservative significance criterion (*p‐*values less than 0.01). Nevertheless, the limited sample size implies that both significant and non‐significant findings be interpreted cautiously.

An additional limitation comes from the categorical classification of groups. The DD group in particular is heterogeneous, as children may have developmental delays in one or more domains. Some of these children may go on to receive an ASD diagnosis or a diagnosis of another developmental disability, and others may not. Future research would benefit from more continuous and nuanced characterization of children's profiles, including their abilities, support needs, and ASD symptoms, across all groups to understand how individual differences relate to social engagement with teachers and peers. Similarly, investigating demographic characteristics, such as sex, race, and ethnicity, and their interaction with ASD will help highlight the influential role they may play in preschool social behavior. Finally, in some classes, TD children were present for the full day while children with ASD were only present for half of the day. Future research should investigate the impact of some peers having extended contact on their social preference relative to those with less contact.

Automated data collection is a relatively novel field with limited studies of the validity and reliability of variables derived from such systems. Time spent in social contact has been utilized as a valid measure of social engagement, while the evidence for social approach velocity as an index of social engagement is less established (Altman et al., 2020; Tsou et al., 2024; Veiga et al., 2017). Social contact and social approach velocity proved to be positively associated with one another in the present study, which suggests both measures index social engagement. However, automated data collection methods, such as those used in this study, are limited in their ability to capture qualitative features of the behaviors they are measuring. Our data‐driven approach emphasizes capturing raw movement data without presupposing specific social outcomes—for instance, the social approach velocity metric does not imply completion of social contact. Future research should aim to continue bridging the gap between objective data and the subjective quality of children's social experiences (e.g., see Tsou et al., 2024). Literature on the reliability of measures generated by automated data collection are promising but limited. Further research investigating the validity and reliability of variables derived from automated data collection is warranted.

Future research should consider classroom, child, and structural features of early childhood educational settings such as adult‐child ratio, ASD ratio, activity or routine, intervention approach, classroom culture, and group structure and composition, as these factors may influence students' opportunities for interaction (Brown et al., 1999; Irvin et al., 2013, 2015; Kamps et al., 1991; Reszka et al., 2012). Teacher characteristics (e.g., passively vs. actively engaged), including how teachers work to facilitate interaction (e.g., through seating charts), should also be considered when examining social preference in children with ASD (Boyd et al., 2008; Irvin et al., 2013). Lastly, future investigations should consider structural characteristics when studying social preference in children with ASD, as these can affect the opportunities young children have to interact with others. Structural characteristics include classroom areas (e.g., pretend play vs. books; Irvin et al., 2017, 2021), size, and set‐up of classrooms (Driscoll & Carter, 2004; Twardosz et al., 1974), as well as different contexts within the early childhood educational space (e.g., early childhood playground).

In conclusion, this study sheds an objective light on the everyday patterns of preschoolers' preferential social engagement in inclusion classrooms. We observed that children with ASD exhibited a heightened preference for engaging with teachers over peers compared to their classmates with TD. These findings underscore the crucial role of teachers as facilitators of social interaction in inclusive classroom settings and re‐emphasize the need for classroom‐based interventions that support the peer interactions of children with ASD.

## FUNDING INFORMATION

The project was funded by the National Science Foundation [IBSS‐L‐1620294, 2150830], the Institute of Education Sciences [R324A180203] and Spencer Foundation, the National Institute on Deafness and Communication Disorders [R01DC018542], and the Simons Foundation Autism Research Initiative [SFI‐AR‐HUMAN‐00004115‐01].

## CONFLICT OF INTEREST STATEMENT

The authors declare no conflicts of interest.

## ETHICS STATEMENT

Recruitment and procedures were approved by the University of Miami's Institutional Review Board (20160509).

## Supporting information


**Data S1:** Supporting Information.

## Data Availability

The data that support the findings of this study are available on the Open Science Framework (https://osf.io/xj9ge/?view_only=6c44665594ce44508ee5e54cd104b75e).
