# Supplementary material for: Children's social preference for teachers versus peers in autism inclusion classrooms: An objective perspective
Source: Autism Res. 2024 Dec 3;18(1):179–94. doi: 10.1002/aur.3276 (PMC11782723; doi:10.1002/aur.3276)
Supplement: Supplementary file 1 — Data S1: Supporting Information. [file AUR-18-179-s001.docx]

**Supplementary Materials**

***Supplemental Table 1:*** *Results from all models*


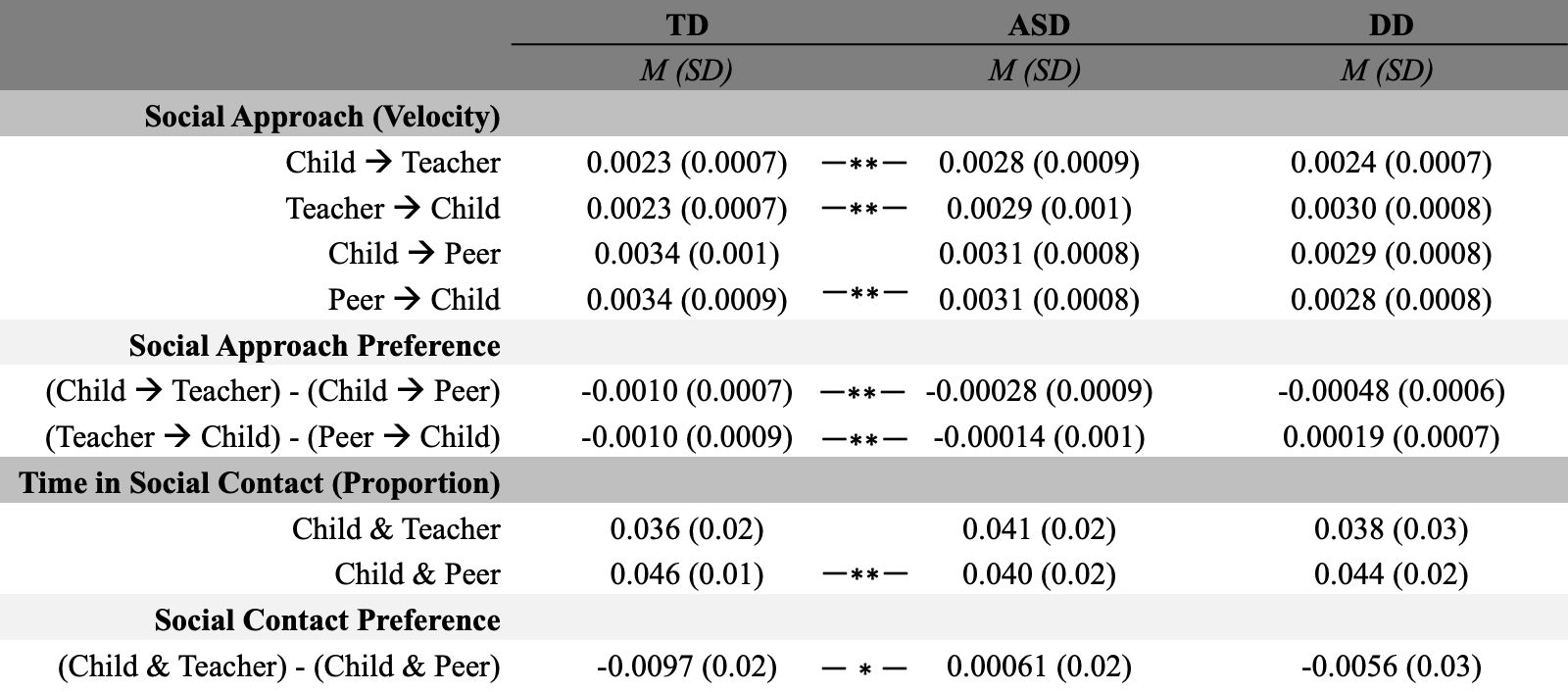


Note: Asterisks indicate significance at the .001 (*) and <.001 (**) levels.

***Supplemental Figure 1:*** *Preliminary results from interaction models.*

| ***Supplemental Figure 1a:*** *Child to peer approach velocity as a predictor of child to teacher approach velocity by eligibility group* | | | | | | | |
| --- | --- | --- | --- | --- | --- | --- | --- |
| **Fixed Effects** | | | | | | | |
| ***Predictors*** | ***B*** | ***SE*** | | ***95% CI*** | ***t*** | ***p*** | ***d*** |
| Intercept | 0.0024 | 0.00019 | | 0.0020 – 0.0027 | 12.28 | <0.001 |  |
| Peer Approach | 0.34 | 0.071 | | 0.20 – 0.48 | 4.83 | <0.001 | 0.60 |
| Peer Approach [ASD] | 0.00047 | 0.00010 | | 0.00027 – 0.00066 | 4.69 | **<0.001** | 1.04 |
| Peer Approach [DD] | 0.00026 | 0.00013 | | 0.00 – 0.00052 | 1.99 | **0.048** | 0.40 |
| Peer Approach*ASD | 0.10 | 0.12 | | -0.14 – 0.34 | 0.84 | 0.404 | 0.11 |
| Peer Approach*DD | 0.17 | 0.14 | | -0.11 – 0.45 | 1.19 | 0.233 | 0.15 |
| **Random Effects** | | | | | | | |
| σ^2^ | 2.56 x 10^-7^ | | 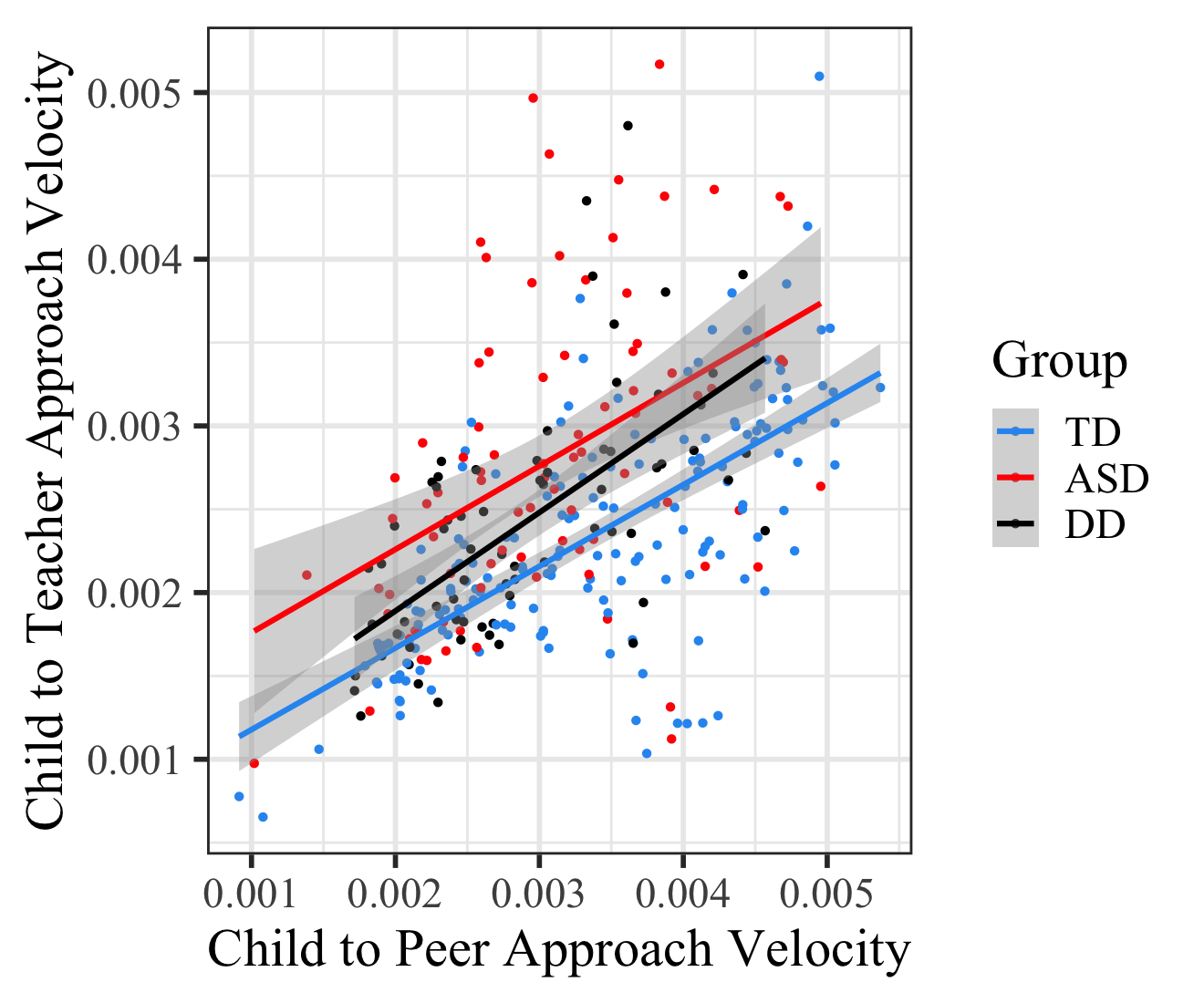 | | | | |
| τ_00_ _subject:classroom_ | 7.01 x 10^-8^ | |  |  |  |  |  |
| τ_00_ _classroom_ | 2.59 x 10^-7^ | |  |  |  |  |  |
| ICC_subject_ | 0.12 | |  |  |  |  |  |
| ICC_classroom_ | 0.51 | |  |  |  |  |  |

Note: All predictor variables were centered at the child-level. TD was the reference group.

| ***Supplemental Figure 1b****: Peer to child approach velocity as a predictor of teacher to child approach velocity by eligibility group* | | | | | | | |
| --- | --- | --- | --- | --- | --- | --- | --- |
| **Fixed Effects** | | | | | | | |
| ***Predictors*** | ***B*** | ***SE*** | | ***95% CI*** | ***t*** | ***p*** | ***d*** |
| Intercept | 0.0026 | 0.00022 | | 0.0022 – 0.0030 | 11.71 | <0.001 |  |
| Peer Approach | 0.086 | 0.093 | | -0.097 – 0.27 | 0.92 | 0.357 | 0.12 |
| Peer Approach [ASD] | 0.00047 | 0.00009 | | 0.00028 – 0.00065 | 4.95 | **<0.001** | 1.13 |
| Peer Approach [DD] | 0.00020 | 0.00013 | | -0.000050 – 0.00045 | 1.54 | 0.124 | 0.31 |
| Peer Approach*ASD | 0.18 | 0.16 | | -0.13 – 0.50 | 1.14 | 0.25 | 0.15 |
| Peer Approach*DD | 0.24 | 0.17 | | -0.086 – 0.56 | 1.45 | 0.149 | 0.19 |
| **Random Effects** | | | | | | | |
| σ^2^ | 3.34 x 10^-7^ | | 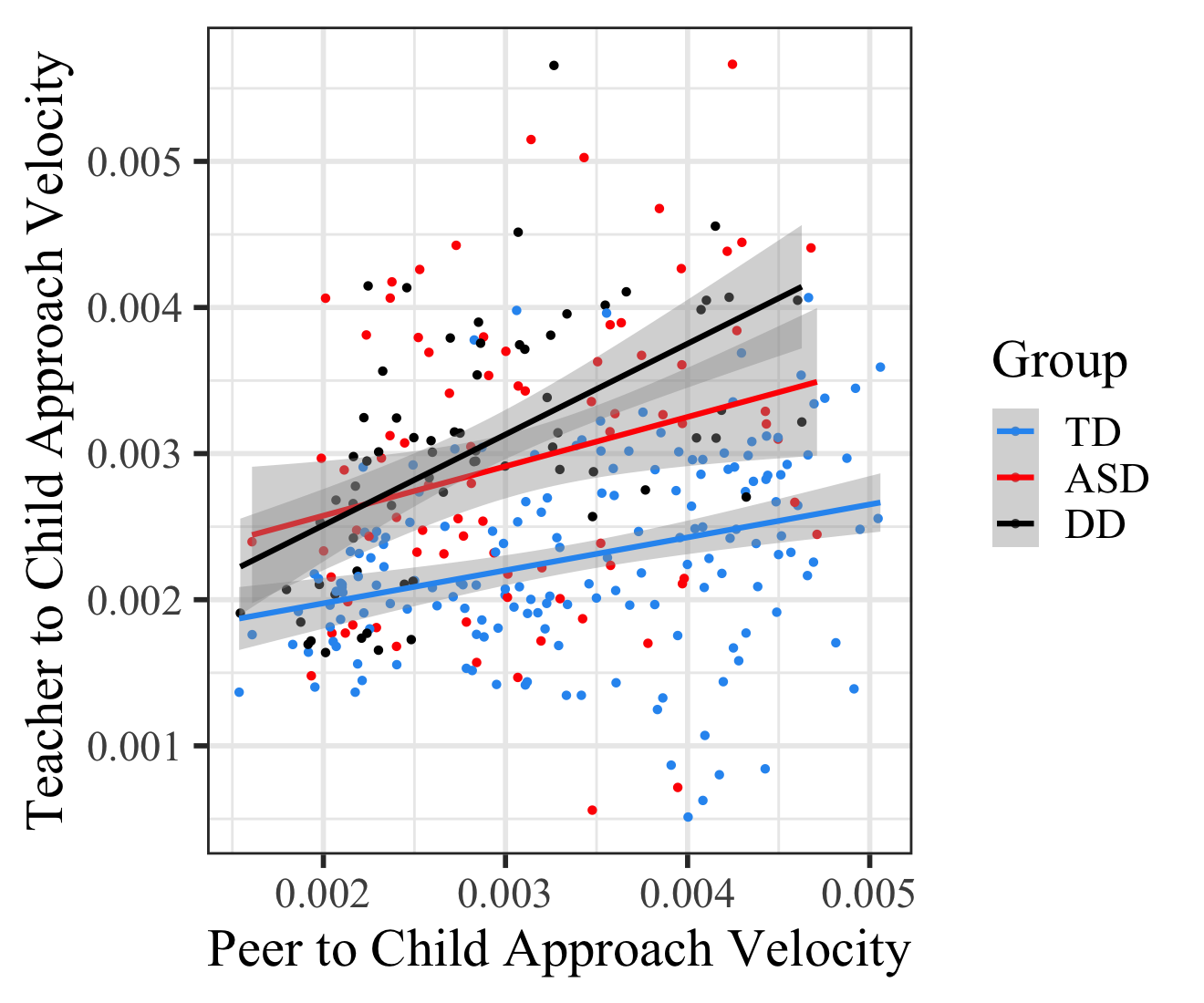 | | | | |
| τ_00_ _subject:classroom_ | 3.44 x 10^-8^ | |  |  |  |  |  |
| τ_00_ _classroom_ | 3.57 x 10^-7^ | |  |  |  |  |  |
| ICC_subject_ | 0.05 | |  |  |  |  |  |
| ICC_classroom_ | 0.49 | |  |  |  |  |  |

Note: All predictor variables were centered at the child-level. TD was the reference group.

| ***Supplemental Figure 1c****: Peer social contact as a predictor of teacher social contact by eligibility group* | | | | | | | |
| --- | --- | --- | --- | --- | --- | --- | --- |
| **Fixed Effects** | | | | | | | |
| ***Predictors*** | ***B*** | ***SE*** | | ***95% CI*** | ***t*** | ***p*** | ***d*** |
| Intercept | 0.038 | 0.0034 | | 0.031 – 0.045 | 11.33 | **<0.001** |  |
| Peer Social Contact | 0.35 | 0.12 | | 0.10 – 0.59 | 2.81 | **0.005** | **0.35** |
| Peer Social Contact [ASD] | 0.0042 | 0.0028 | | -0.0013 – 0.0097 | 1.50 | 0.135 | **0.36** |
| Peer Social Contact [DD] | 0.0016 | 0.0037 | | -0.0056 – 0.0088 | 0.43 | 0.665 | **0.09** |
| Peer Social Contact*ASD | -0.24 | 0.23 | | -0.69 – 0.21 | -1.07 | 0.285 | -0.14 |
| Peer Social Contact*DD | 0.32 | 0.24 | | -0.16– 0.80 | 1.30 | 0.194 | 0.17 |
| **Random Effects** | | | | | | | |
| σ^2^ | 3.75 x 10^-4^ | | 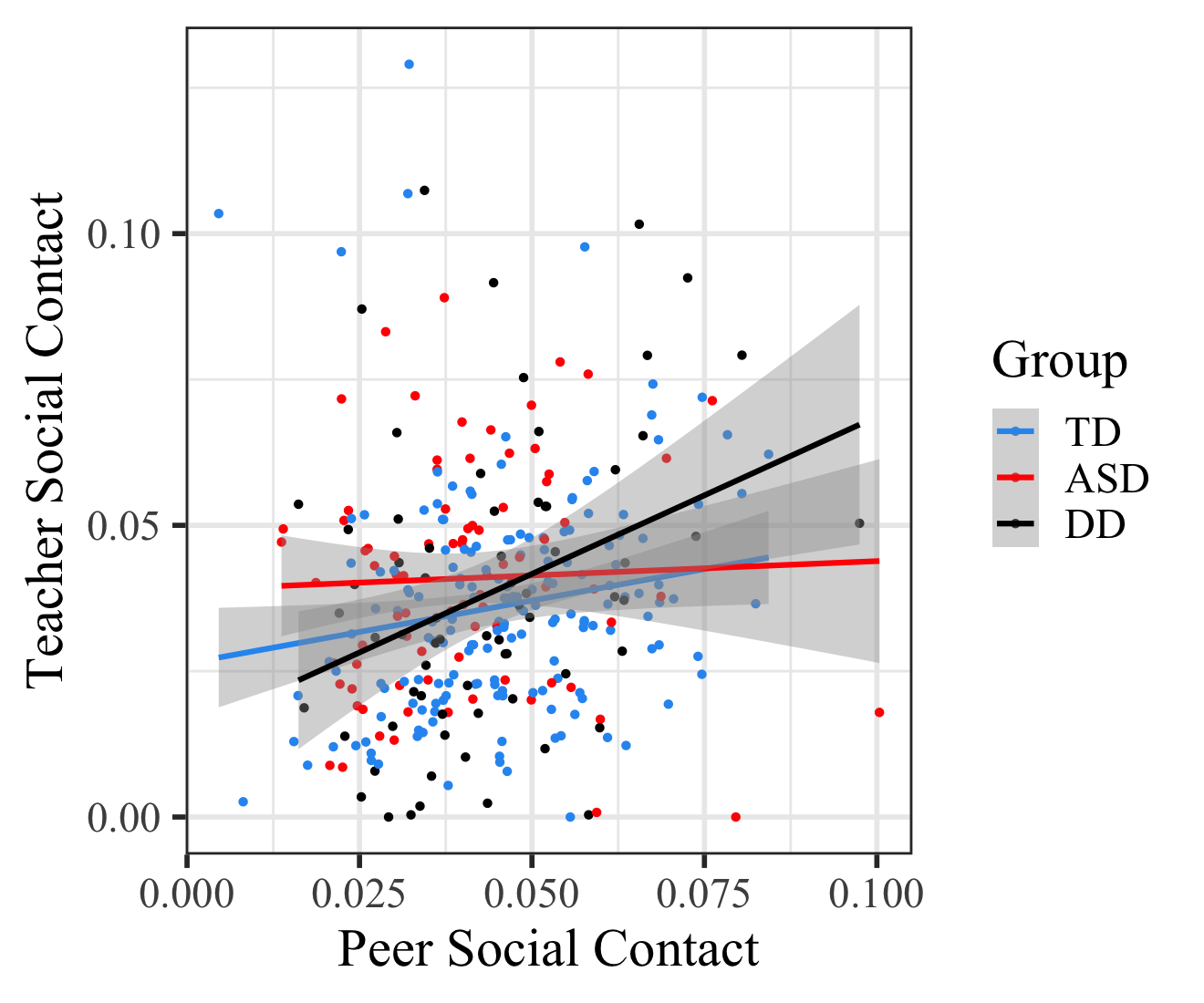 | | | | |
| τ_00_ _subject:classroom_ | 1.00 x 10^-5^ | |  |  |  |  |  |
| τ_00_ _classroom_ | 6.30 x 10^-5^ | |  |  |  |  |  |
| ICC_subject_ | 0.02 | |  |  |  |  |  |
| ICC_classroom_ | 0.14 | |  |  |  |  |  |

Note: All predictor variables were centered at the child-level. TD was the reference group.

| ***Supplemental Figure 1d****: Child to peer approach velocity as a predictor of peer social contact by eligibility group* | | | | | | | |
| --- | --- | --- | --- | --- | --- | --- | --- |
| **Fixed Effects** | | | | | | | |
| ***Predictors*** | ***B*** | ***SE*** | | ***95% CI*** | ***t*** | ***p*** | ***d*** |
| Intercept | 0.049 | 0.0031 | | 0.043 – 0.055 | 15.87 | **<0.001** |  |
| Peer Approach | 7.08 | 1.68 | | 3.77 – 10.38 | 4.21 | **<0.001** | **0.48** |
| Peer Approach [ASD] | -0.0071 | 0.0017 | | -0.010 – -0.0037 | -4.12 | **<0.001** | **-0.46** |
| Peer Approach [DD] | -0.0046 | 0.0023 | | -0.0092 – -0.000080 | -2.00 | **0.046** | **-0.23** |
| Peer Approach*ASD | -3.26 | 2.96 | | -9.09 – 2.57 | -1.10 | 0.272 | -0.12 |
| Peer Approach*DD | 1.56 | 3.46 | | -5.26 – 8.37 | 0.45 | 0.653 | 0.05 |
| **Random Effects** | | | | | | | |
| σ^2^ | 1.52 x 10^-4^ | | 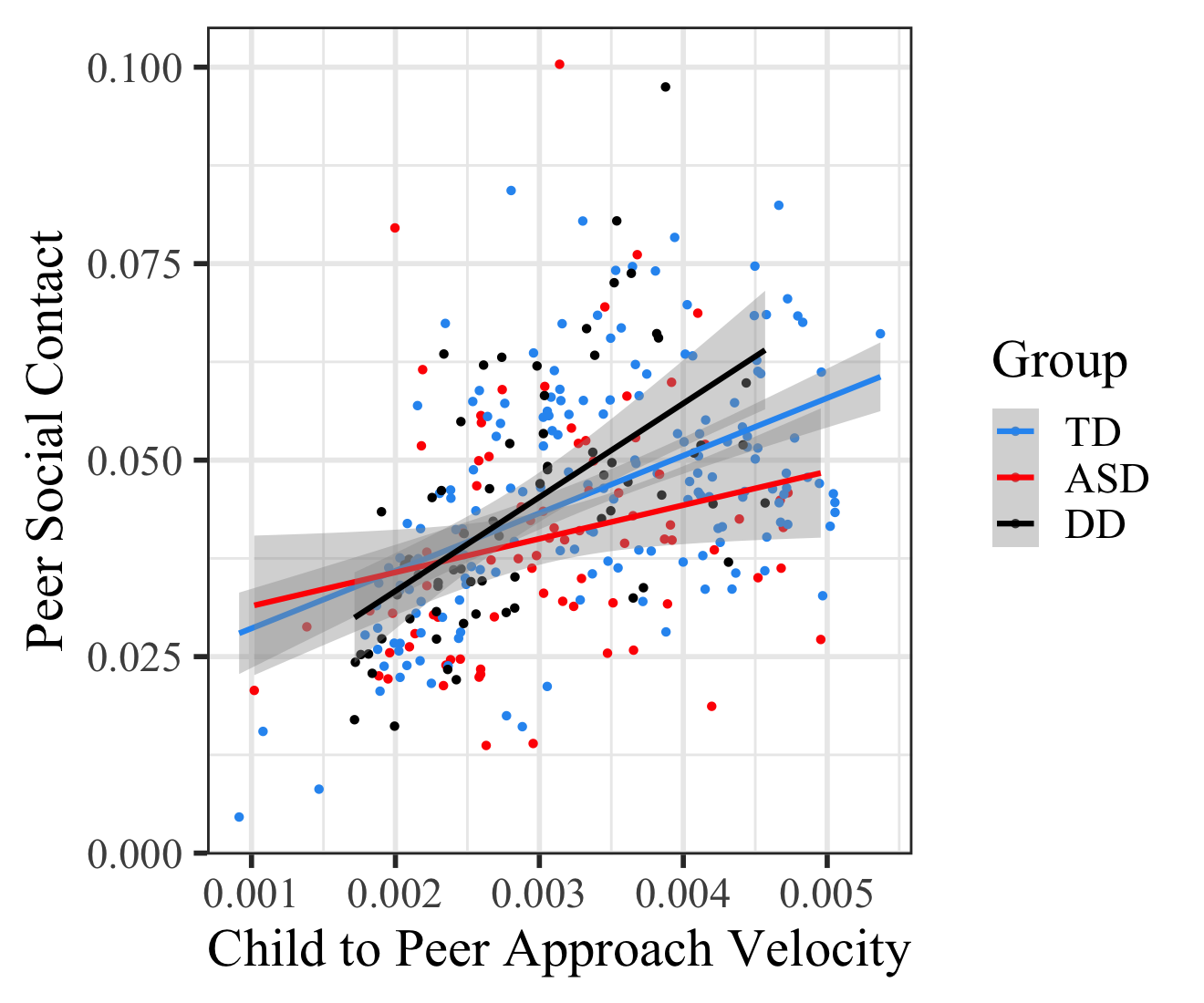 | | | | |
| τ_00_ _subject:classroom_ | 0.00 | |  |  |  |  |  |
| τ_00_ _classroom_ | 6.47 x 10^-5^ | |  |  |  |  |  |
| ICC_subject_ | 0.00 | |  |  |  |  |  |
| ICC_classroom_ | 0.30 | |  |  |  |  |  |

Note: All predictor variables were centered at the child-level. TD was the reference group.

| ***Supplemental Figure 1e****: Child to teacher approach velocity as a predictor of teacher social contact by eligibility group* | | | | | | | |
| --- | --- | --- | --- | --- | --- | --- | --- |
| **Fixed Effects** | | | | | | | |
| ***Predictors*** | ***B*** | ***SE*** | | ***95% CI*** | ***t*** | ***p*** | ***d*** |
| Intercept | 0.038 | 0.0033 | | 0.031 – 0.044 | 11.49 | **<0.001** |  |
| Teacher Approach | 6.95 | 3.24 | | 0.57 – 13.33 | 2.14 | **0.033** | **0.26** |
| Teacher Approach [ASD] | 0.0044 | 0.0028 | | -0.0012 – 0.0099 | 1.55 | 0.122 | **0.37** |
| Teacher Approach [DD] | 0.0017 | 0.0037 | | -0.0055 – 0.0089 | 0.47 | 0.642 | **0.10** |
| Teacher Approach*ASD | 2.27 | 4.84 | | -7.25 – 11.79 | 0.47 | 0.639 | 0.06 |
| Teacher Approach*DD | 10.74 | 6.42 | | -1.88 – 23.37 | 1.67 | 0.095 | 0.22 |
| Random Effects | | | | | | | |
| σ^2^ | 2.56 x 10^-7^ | | 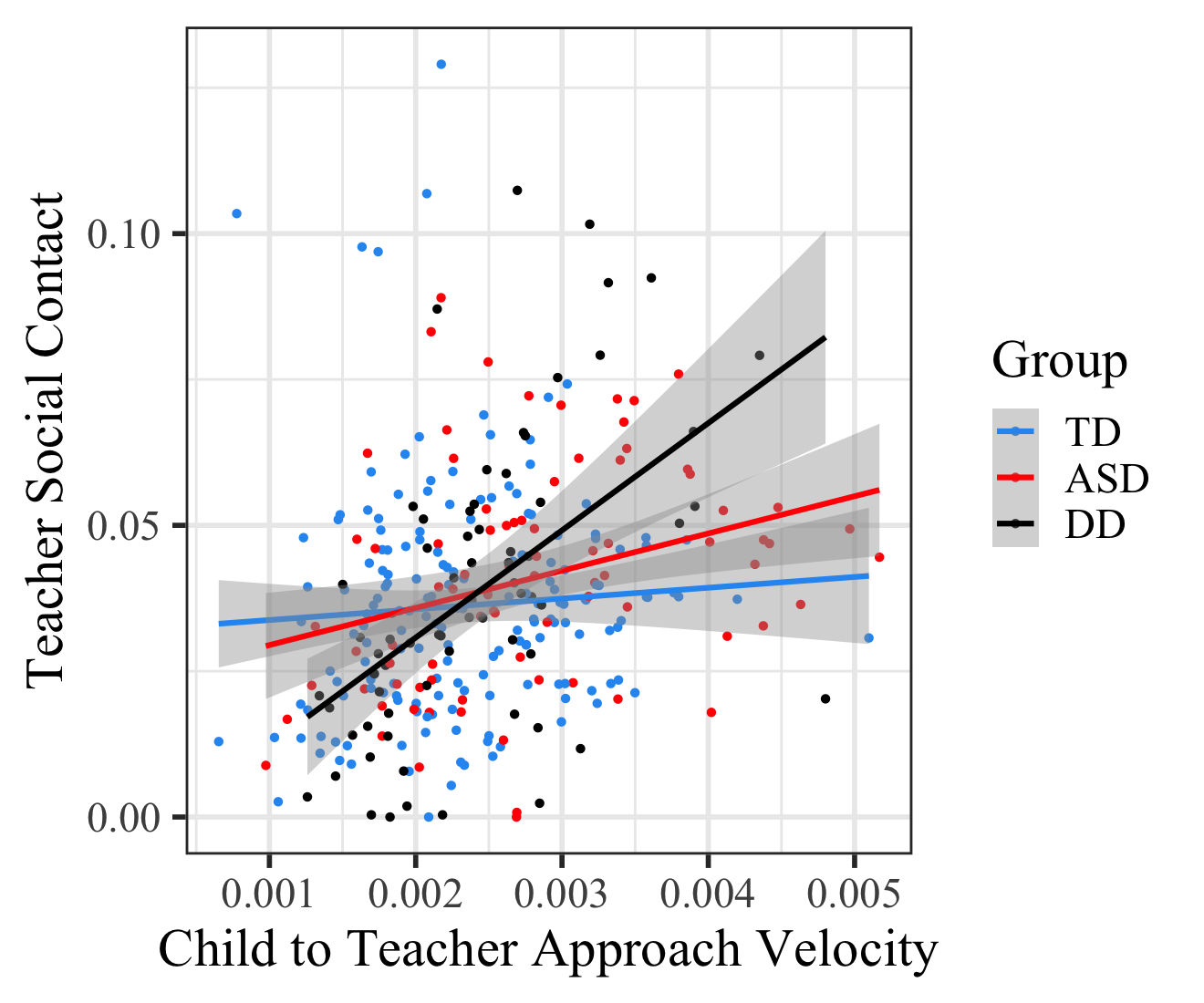 | | | | |
| τ_00_ _subject:classroom_ | 7.01 x 10^-8^ | |  |  |  |  |  |
| τ_00_ _classroom_ | 2.59 x 10^-7^ | |  |  |  |  |  |
| ICC_subject_ | 0.03 | |  |  |  |  |  |
| ICC_classroom_ | 0.14 | |  |  |  |  |  |

Note: All predictor variables were centered at the child-level. TD was the reference group.
